# Fully automated whole-liver volume quantification on CT-image data: Comparison with manual volumetry using enhanced and unenhanced images as well as two different radiation dose levels and two reconstruction kernels

**DOI:** 10.1371/journal.pone.0255374

**Published:** 2021-08-02

**Authors:** Florian Hagen, Antonia Mair, Michael Bitzer, Hans Bösmüller, Marius Horger

**Affiliations:** 1 Department of Diagnostic and Interventional Radiology, Eberhard-Karls-University, Tübingen, Germany; 2 Department of Internal Medicine I, University Hospital Tübingen, Tübingen, Germany; 3 Department of Pathology and Neuropathology, University Hospital Tübingen and Eberhard Karls University Tübingen, Tübingen, Germany; Sun Yat-Sen University, CHINA

## Abstract

**Objectives:**

To evaluate the accuracy of fully automated liver volume quantification vs. manual quantification using unenhanced as well as enhanced CT-image data as well as two different radiation dose levels and also two image reconstruction kernels.

**Material and methods:**

The local ethics board gave its approval for retrospective data analysis. Automated liver volume quantification in 300 consecutive livers in 164 male and 103 female oncologic patients (64±12y) performed at our institution (between January 2020 and May 2020) using two different dual-energy helicals: portal-venous phase enhanced, ref. tube current 300mAs (CARE Dose4D) for tube A (100 kV) and ref. 232mAs tube current for tube B (Sn140kV), slice collimation 0.6mm, reconstruction kernel I30f/1, recon. thickness of 0.6mm and 5mm, 80–100 mL iodine contrast agent 350 mg/mL, (flow 2mL/s) and unenhanced ref. tube current 100mAs (CARE Dose4D) for tube A (100 kV) and ref. 77mAs tube current for tube B (Sn140kV), slice collimation 0.6mm (kernel Q40f) were analyzed. The post-processing tool (*syngo*.CT Liver Analysis) is already FDA-approved. Two resident radiologists with no and 1-year CT-experience performed both the automated measurements independently from each other. Results were compared with those of manual liver volume quantification using the same software which was supervised by a senior radiologist with 30-year CT-experience (ground truth).

**Results:**

In total, a correlation of 98% was obtained for liver volumetry based on enhanced and unenhanced data sets compared to the manual liver quantification. Radiologist #1 and #2 achieved an inter-reader agreement of 99.8% for manual liver segmentation (p<0.0001). Automated liver volumetry resulted in an overestimation (>5% deviation) of 3.7% for unenhanced CT-image data and 4.0% for contrast-enhanced CT-images. Underestimation (<5%) of liver volume was 2.0% for unenhanced CT-image data and 1.3% for enhanced images after automated liver volumetry. Number and distribution of erroneous volume measurements using either thin or thick slice reconstructions was exactly the same, both for the enhanced as well for the unenhanced image data sets (p> 0.05).

**Conclusion:**

Results of fully automated liver volume quantification are accurate and comparable with those of manual liver volume quantification and the technique seems to be confident even if unenhanced lower-dose CT image data is used.

## Introduction

Precise assessment of liver volume is required in many clinical settings. In particular, in patients admitted for liver surgery (liver lobe resection or liver transplantation), estimation of the future liver volume remnant as well as of the donor organ size is essential for good patient management and for reducing the risk of postoperative hepatic insufficiency [1, 2]. Of interest is also the loss of liver volume during chronic liver disorders (e.g. cirrhosis) as well as after iatrogenic liver ischemia with rapid deterioration of liver function. Moreover, liver volumetry has been increasingly performed preceding intra-arterial or portal-venous interventions [3–6]. Due to its complex, variable shape, and proximity to other anatomical structures, liver segmentation, and quantification is challenging. Volumetry of the liver is by now performed using different techniques which generally rely on different segmentation techniques, contour recognition, etc. [7–9]. Manual contour tracing for organ volumetry is knowingly limited as it is cumbersome, time-consuming and underlies inter- and intra-observer variability [10]. For this reason, semi-automated and automated liver volume calculation software tools have been further developed and are now in use worldwide [8–11]. However, differences exist between post-processing tools, many of them showing expectedly strengths, but also limitations by comparison with the manual quantification [2, 9, 12, 13]. Moreover, the dependency of these measurements from CT-examinational protocol (i.e. unenhanced vs. contrast-enhanced CT; contrast volume and enhancement phase as well as slice thickness) are still an issue of debate in searching for optimal solution [14, 15].

In light of emerging new AI-based organ detection and volume quantification techniques we set out to evaluate already available, FDA-approved software made available by the vendor. For this purpose we used already existent CT-image data sets (retrospective design) of oncologic patients referred for staging or monitoring purposes at our institution which were all acquired using standardized examinational protocols. They consisted of both reduced-dose unenhanced submillimeter as well as contrast-enhance thin-slice (submillimeter) CT-images obtained in the portal-venous phase.

So, the purpose of this study was to find out if the fully-automated available software delivers accurate volumetric quantification of the liver compared with the ground truth and if even reduced-dose unenhanced image data obtained with different collimation kernels would be comparably suitable for accomplishing this task.

## Materials and methods

### Patient characteristics

This retrospective data evaluation was approved by the institutional review board which was assigned the approval number 841-2020-B02. Verbal and written informed consent was waived due to the retrospective nature of the study.

Between January 2020 and May 2020 we retrospectively evaluated a total of 300 livers of 267 consecutive oncologic patients who were referred to staging or treatment monitoring purposes to our radiology department undergoing standardized CT-examinations (inclusion criteria). We also included double CT-examinations of 33 patients who fulfilled the inclusion criteria. Patients undergoing CT-examinations using other imaging protocols (e.g. no thin-slice image data, different enhancement phases, different contrast agent volumes) were excluded from the final analysis (n = 22).

### CT-examinational protocol

All CT-examinations were performed on a 256-slice dual-energy CT-scanner (Siemens Definition Flash; Forchheim, Germany). All patients underwent first unenhanced dual-energy CT using ref. tube current 100mAs (CARE Dose4D) for tube A (100 kV) and ref. 77mAs tube current for tube B (Sn140kV), 0.6 mm single collimation width, table speed of 70 cm/s, table feed per rotation of 23 cm, spiral pitch factor 0.6, matrix 512 x 512 and a reconstruction kernel Q40f. Subsequently, contrast-enhanced CT in the portal-venous phase was performed using 80–100 mL contrast agent volume (Imeron 350, BRACCO Imaging Germany GmbH), a flow of 2mL/s, same slice thickness and collimation parameters as for unenhanced helical using ref. tube current 300mAs (CARE Dose4D) for tube A (100 kV) and ref. 232mAs tube current for tube B (Sn140kV) and reconstruction kernel I 30f/1. Mean "Computed Tomography Dose Index per Volume" (CTDI_Vol_) was 2.824 mGy for unenhanced and 10.286 mGy for enhanced image data sets. A sinogram affirmed iterative reconstruction (SAFIRE) at level1 was used.

### CT-image data analysis

All image data were transferred to the *syngo*.via server (VB 30) for post-processing.

### Manual liver volume measurements

Manual liver volume quantification was performed using a post-processing tool called *syngo*.CT Liver Analysis. This allows for manual drawing of liver contours slice-by-slice (at least at ten different levels, every 10–15 mm in craniocaudal direction) followed by semiautomatic interpolation and contour generation). In case of faulty contour drawing, manual correction in a slice-by-slice manner had to be performed. The pre-step of vessel detection (depending on the enhancement phase) was skipped as we aimed in this study solely at volumetric quantification of the entire liver. Only enhanced (mixed series) and thin slice (0.6mm) image data sets were used for manual liver volume measurements by both radiologists. The results of manual liver volume quantification were archived and reappraised by the senior radiologist (30 years of experience) and only the accurate measurements were accepted as ground truth.

For this purpose, the senior radiologist had to reevaluate all the results of the manual volumetry throughout all levels assuring correct encompassment of the liver contours, readjusting them if necessary.

Both the senior radiologist and the resident radiologists were unaware of the results of automated liver volume calculation.

### Automated liver volume measurements

The automated liver volumetry was applied to both thin (0.6 mm) and thick slice data sets (5mm), whether enhanced or unenhanced.

**Fig 1:**
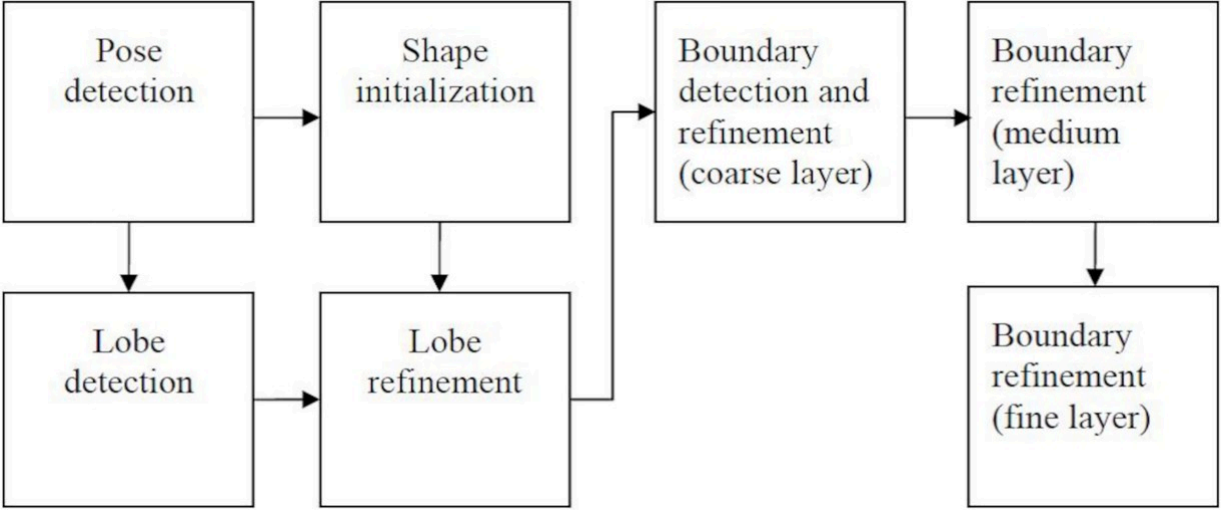
Flowchart and components of the liver segmentation system.

The main steps in the calculation of automated liver volumes are shown by the chart below (Fig 1).

Our software uses a hierarchical shape model of the liver created using off-line training. To reduce the size of the searching space for the nine parameters [p = (p1, p2, p3), θ = (θ1, θ2, θ3), s = (s1, s2, s3)], required for the best pose of the liver, marginal space learning (MSL) was used. MSL intuitively reduces the size of the searching space by marginal space inference and sequentially propagating to the whole space. In our case, the 9D parameter space was decomposed into three marginal spaces. To learn the marginal probabilities (i.e., Pr(p|vol), Pr(θ|p, vol), and Pr(s|θ, p, vol)), the probabilistic boosting tree (PBT) was used. The detected shape is described by the scaling, rotation, and translation parameters. For shape initialization and further refinement, a principal component analysis shape model was used.

The output of the shape initialization module was then further fed into the lobe refinement module. Previous methods mostly approximated the boundary response by simply checking gradients or intensity distribution along organ surfaces. However, the information gathered this way is not enough for our task because the texture pattern of livers has a large variability. When dealing with data from different scanning protocols, the variability is even larger. To address this problem, we learned Pr(bdry|q, voll) using PBT and steerable features. Besides, the spherical coordinates of mesh points were included as features. These coordinates provided important distinctive information because the intensity patterns around the boundary are closely tied to their positions on the liver surface. The heterogeneity of texture pattern along liver boundaries suggests the use of patch dependent boundary classifiers. To this end, we decomposed the liver surface to five patches: liver-lung, liver-heart, liver-kidney, liver-tissue, and liver-misc. At the same time as the boundary detection was running, the mesh at the coarsest layer was refined. Then the mesh at the coarsest layer was upsampled to a medium resolution layer and refined again. The procedure continued till the finest layer was reached. For the up sampling between layers, the thin plate spline (TPS) warping was used. Given two point sets with correspondence between them, TPS found a nonlinear warping by minimizing second-order “bending energy”. In our task, mesh points at a coarse level correspond to a subset of mesh points.

The automated liver volume quantification was performed by the same two residents (A.M.) and (H.F.) with 1-month and 1-year of experience in abdominal and liver imaging.

Oversegmented various locations were all registered for subsequent analysis. The automated liver volume quantification was applied on both image data (enhanced and unenhanced) with known differences with respect to the applied energy level and reconstruction kernel.

### Statistical analysis

Statistical analysis was performed using SPSS (version 27.0.0, IBM, Armonk, NY, USA). Continuous data were expressed as means ± standard deviation (SD). Agreement for liver volumetry for both readers, and for the different slice thicknesses as well as separately for the enhanced and unenhanced datasets was tested by Kronbach’s intraclass correlation coefficient (ICC). After verification of Gaussian distribution, liver volume differences, as well as differences in the number of pitfalls were analyzed by a paired t-test. Differences in liver volume measurements for the thin slice reconstructions were displayed in Bland-Altman-Plots. Statistical significance was set for p ≤ 0.05.

## Results

### Patient characteristics

Mean age of the 164 male patients was 64 ± 12 years and 64 ± 13 years for the 103 female patients. In total, 12.9% (38/300) of examined livers revealed malignant lesions whereas 25.3% presented with benign hepatic lesions (Table 1).

**Table 1:** Patient characteristics.

		Total (n = 300)
Age [mean ± standard deviation]		64 ± 12 years
Sex [n],(%)	Male	164 (61.4%)
	Female	103 (38.6%)
Liver metastases [n]		38 (12.9%)
Benign changes of the liver parenchyma [n]		76 (25.3%)
Cysts		60 (20.0%)
Hemangioma		9 (3.0%)
Calcification		4 (1.3%)
Adenoma and focal nodular hyperplasia		3 (1.0%)

### Interrater-agreement for the first 100 manual liver segmentations in thin slice enhanced image data sets

**Fig 2:**
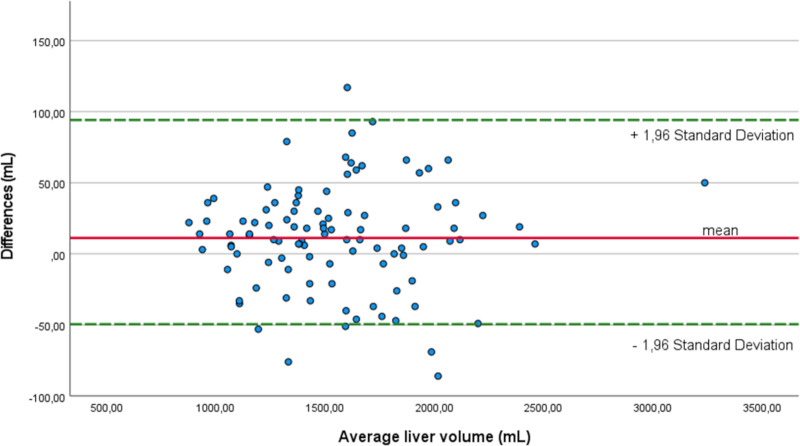
Bland-Altman-Plot for radiologist #1 and #2.

Mean liver volume after manual segmentation of radiologist #1 was 1554 ± 39 mL and 1543 ± 39 mL for radiologist #2, corresponding to an ICC of 0.998 (Fig 2).

### Automated vs. manual liver segmentation

**Fig 3:**
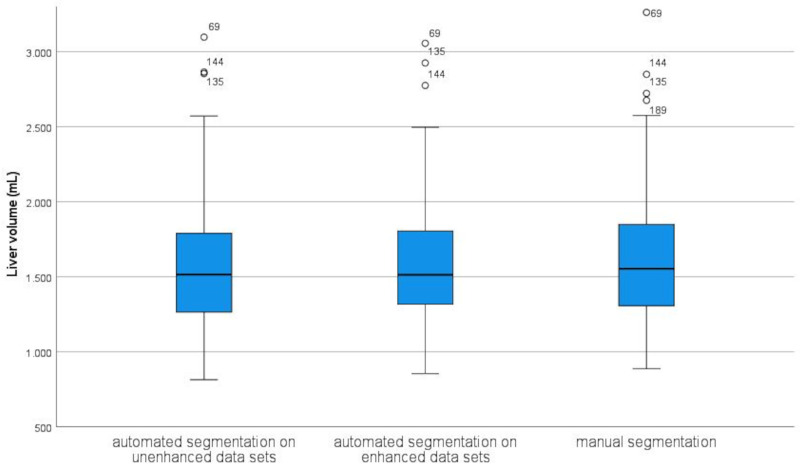
Mean liver volume analysis for thin slice image data sets.

The automated liver segmentation based on thin slice enhanced CT-image data sets yielded a mean liver volume of 1568 ± 382 mL [IQ: 1513 mL, SW: 2201 mL] whereas for the thin slice unenhanced CT-image data sets, the tool calculated a mean volume of 1551 ± 398 mL [IQ: 1515 mL, SW: 2283 mL]. In comparison, 1573 mL [IQ: 1520 mL, SW: 2211 mL] volume was measured on the basis of the thick slices in enhanced image data sets and 1546 mL [IQ: 1499 mL, SW: 2314 mL] in unenhanced image data sets, corresponding to an ICC of 0,997 and 0,998, respectively. The ground truth liver volumetry performed under supervision of the senior radiologist yielded a mean liver volume of 1592 ± 403 mL [IQ: 1553 mL, SW: 2374 mL]. Four patients (Fig 3) presented with considerable hepatomegaly due to fatty liver (n = 2) (ID-n° 69 and 189), acute lymphocytic leukemia (n = 1) (ID-n° 135) and mantle cell lymphoma (n = 1) (ID-n° 144) resulting in values above the standard deviation (boxplot outliers).

**Fig 4:**
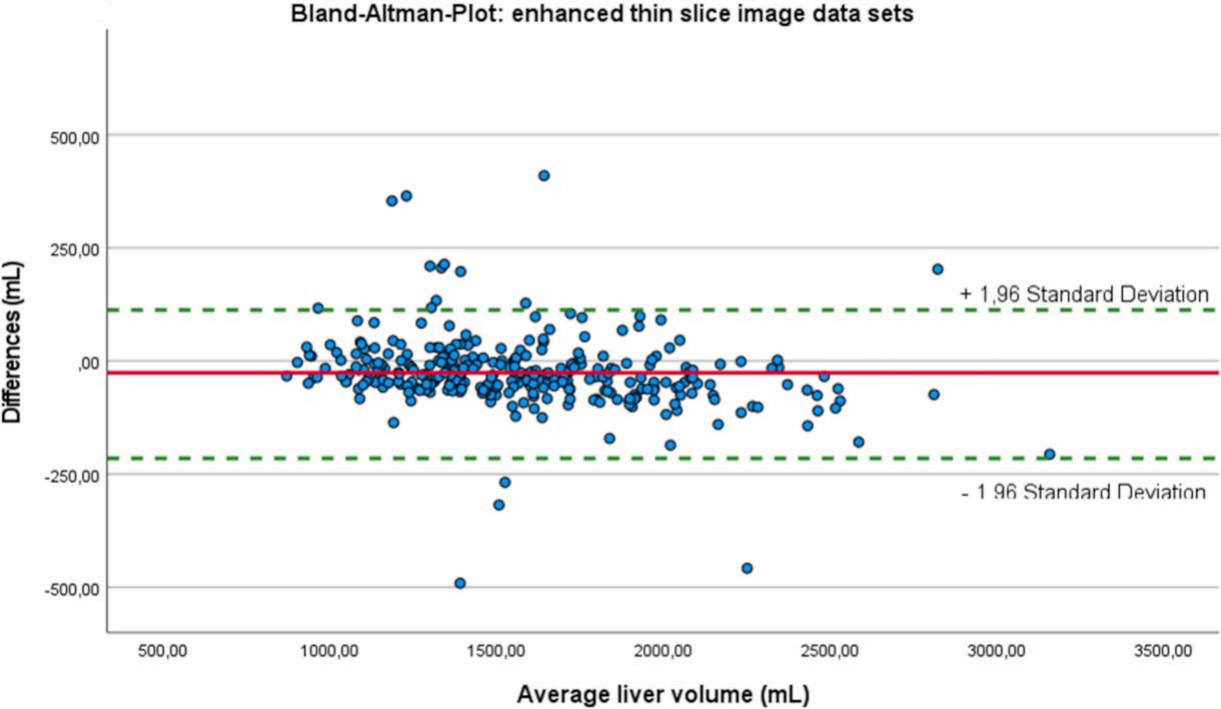
Correlation between manual and automated liver segmentation for enhanced image data sets.

**Fig 5:**
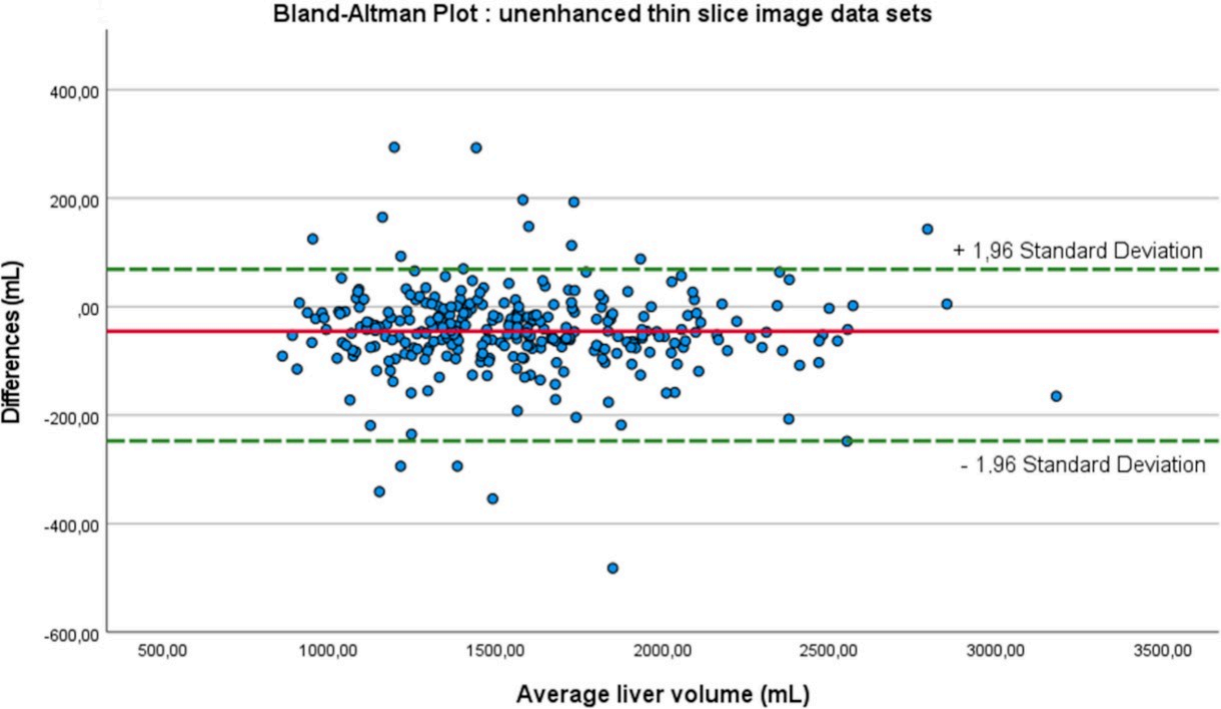
Correlation between manual and automated liver segmentation for unenhanced image data sets.

ICC between results of manual and automated liver volumetry based on thin slice enhanced CT-image data as well as for thin slice unenhanced CT-image data was 0.975 and 0.974, respectively (Figs 4 and 5). The liver volumetries for thin slice enhanced and unenhanced image data sets lead to a straight line equation of 88.53 + 0.93 * X and 1.78 + 0.97 * X, respectively. The intersection is at 2104 mL for both automated calculated liver volumes. Above this value, the calculated liver volume from enhanced image data sets differed more than 3.4% from the manually measured liver volume (Fig 4) compared to the unenhanced image data sets (2.0%, Fig 5) (p = 0.035). Below the intersectional point of 2104 mL, the automated calculated liver volume underestimated the liver volume by an average of 1.4% compared to 3.0% for the unenhanced image data sets (p = 0.000).

### Pitfalls of the automated liver segmentation in enhanced and unenhanced data sets

**Fig 6:**
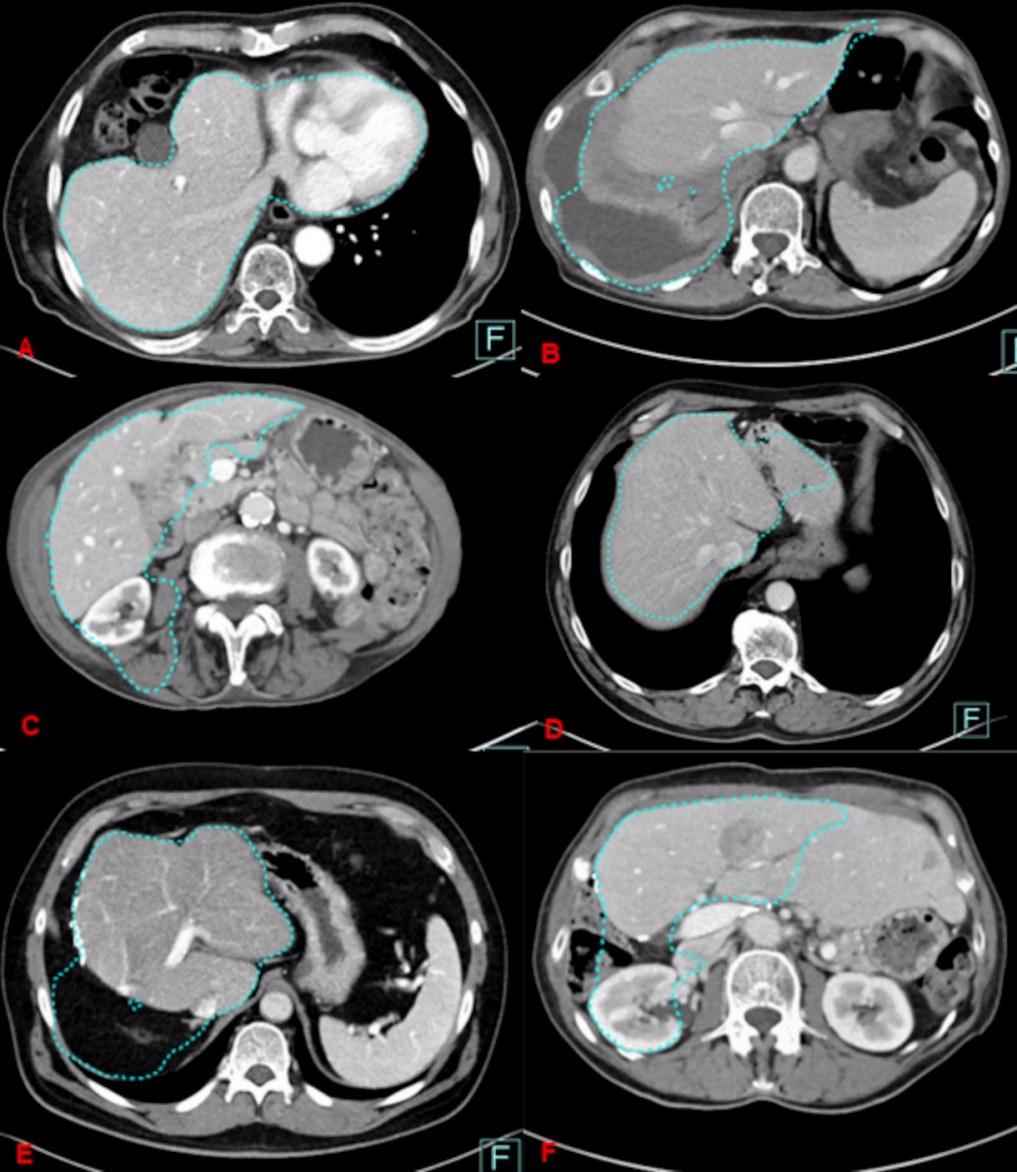
Pitfalls for enhanced image data sets.

The number and distribution of pitfalls was exactly the same between thin and thick slice reconstructions, both for the enhanced as well for the unenhanced image data sets (p> 0.05). A 5% overestimation of the liver volume compared with the ground truth was found for automated enhanced CT-image data in 12 cases and in 11 cases for unenhanced image data based liver volume quantification (different patients) (Figs 4 and 5). Interestingly, in contrast enhanced data sets, this significant difference is found with increasing liver volume (Fig 4) whereas the mismatch for unenhanced data sets is more often seen in lower liver volumes (Fig 5). For the enhanced image data sets, the program included four times the already removed liver volume after hemi-hepatectomy, four times the heart (total and partially included), two times the right kidney, once the stomach and more times perihepatic fluid (Fig 6A–6E).

Automated liver volume quantification based on unenhanced CT-image data underestimated the liver volume (<5%) compared to the ground truth (n = 6) whereas based on enhanced CT-image data in only in four cases. In the latter, the total liver volume was erroneously calculated too small because of leaving parts of liver parenchyma unincluded in the measurement (Fig 6F).

**Fig 7:**
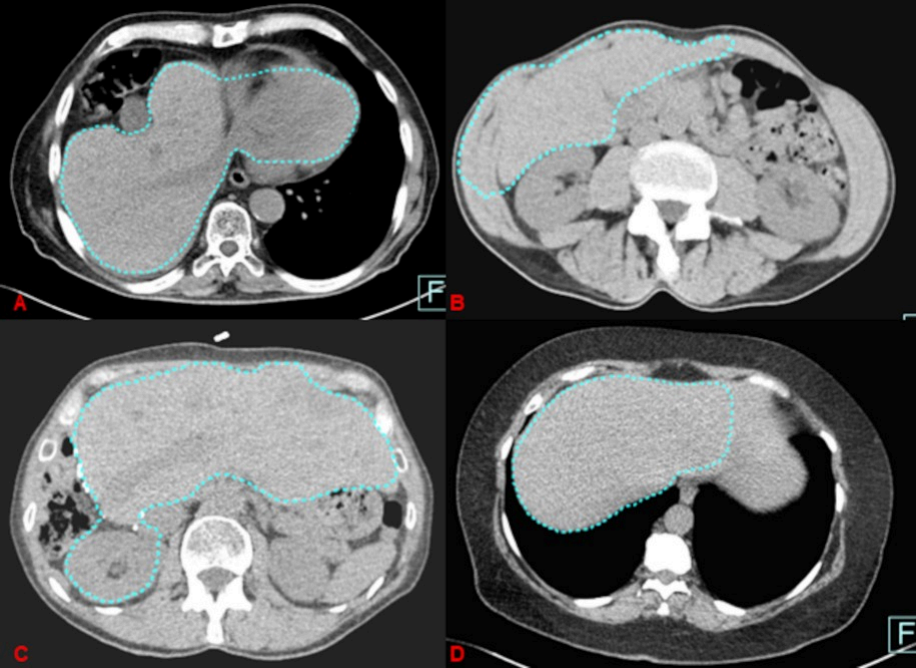
Pitfalls for unenhanced image data sets.

Underestimation of liver volume based on unenhanced CT-image data is shown on Fig 7A–7D.

For the unenhanced image data sets, the automated liver segmentation erroneously included four times the abdominal muscles, once the heart and once the kidney (Fig 7A–7C). In 10 cases, the total liver volume was calculated too small because of skipping liver volume (Fig 7D), leading to a volume change within the standard deviation.

## Discussion

Results of fully automated liver volume quantification using an available FDA-approved software proved accurate showing minimal (mean <2%) variation from the manual liver volumetry which was the ground truth in our study. This statement was true for both imaging protocols used in this study. Moreover, this performance also applied on thick-slice (5mm) based liver volumetry. Of note, results of manual liver volume quantification were in excellent agreement between the two radiology residents.

Overestimation of liver volume by automated software was comparable for reduced-dose unenhanced CT-image data and contrast-enhanced normal-dose image data (12 vs. 11). However, we found slightly more underestimations of liver volume with unenhanced CT-images, regardless of thick or thin slice reconstructions, compared to contrast-enhanced images (6 vs. 4). The latter is presumably due to less accurate delineation of liver contours to the neighbors with unenhanced images whereas similar attenuation values of adjacent organs like heart, kidney, etc. seemed to be responsible for erroneous co-segmentation of parts of these organs leading finally to volume overestimation. All in all, the incidence of erroneous measurements stayed very low and could be quickly detected and easily manually corrected by the reader being considered acceptable if below 5% of the ground truth. The semi-automated post-processing tool used in this study, aims at supporting the whole diagnostic assessment for liver tumor or liver transplantation surgery by guiding through several work steps, starting from whole liver segmentation, vessel tree analysis and liver sub-segmental analysis up to sub-volume analysis of the liver. In our work we focused on the initial workflow step, which achieves semi-automatic segmentation of the whole organ, since this is the most crucial part of the workflow and influencing the latter steps.

This topic has been addressed in many previous reports. Suzuki et al. using an automated 3D active contour segmentation vs. manual segmentation demonstrated that the former was substantially more efficient, however not reaching the desired minimal variation of <3% to the latter method [11]. To exclude differences in the liver volume calculation due to inclusion of the intrahepatic vessels which can be reliably excluded only by the automated volumetry tool as in the work by Suzuki, we deliberately included them in the final quantification. Nakayama et al., in a similar attempt using geodesic active contour segmentation, found a good correlation (r = 0.792) between automated and manual liver volumetry using 2.5 mm reconstruction slice thickness in their study pointing on the considerable reading time reduction (32.8 vs. 4.4 min) with automated techniques [2]. However, their results did not reach the accuracy presented in our work. One major criticism of automated volume quantification in the past was the potential for inaccuracy due to e.g. low attenuation differences between liver parenchyma and neighbor organs leading to volume over- or underestimation. For this reason, semi-automated methods, e.g. random walk, were advocated as they use multiple intuitive user inputs interfering with this process maintaining control on all steps [16]. Many approaches have been developed for organ segmentation, contour detection most of them combining more of these techniques [13, 17, 18].

Whereas many of the technical limitations reported earlier were related to longer acquisition times (non-helical CT era), limitations due to breath-holding and the used breathing phase as well as to slice thickness, enhancement phase, etc. have been solved meanwhile with the advance of rapid acquisition-CT scanner, some other sources of errors linked to e.g. the anatomical properties of the liver are still challenging [19, 20].

Our software using a hierarchical shape model of the liver created using off-line training proved comparably very robust and reliable.

Using high-dose, thin-slice contrast-enhanced portal-venous CT-image data, Luciani et al. undertook liver volume measurements using multi-phase vs. single-phase CT-image data showing that the latter was less correlated (r = 0.76) to the manual quantification [21]. By comparison, in our study, the liver volume quantification based on a single unenhanced, reduced-dose CT-image data yielded excellent results. Similar outcome has been reported using AI-based software with a mean absolute error of 2.3% of the averaged liver volumes [22]. Zahel et al. reported comparable results for total liver volume quantification using CECT image data obtained in the portal-venous phase proposing another software program [23]. Current AI-based efforts are being made for additional liver and other organs segmentation as well as for lesion detection inside these organs and could thus emerge to a new state-of-the art for lesion and organ volumetry [24, 25].

Summarized, we believe that our study which was performed in a larger series of patients is the first to objectively assess the accuracy of liver volumetric measurements using a semi-automated post-processing software as dependent on the slice thickness as well as on the presence/absence of IV contrast agent. While most recent scientific publications in the area of automated organ segmentation often apply AI-based approaches utilizing convolutional neural networks, these approaches are rarely to be found in commercially available applications, due to cost and effort related to achieve regulatory approval of AI-based applications.

Our study has some limitations. First, in this study only one scanner with an own, specific postprocessing software was available so that the conclusions of this study could eventually not fit on all other CT scanners. Second, it was performed in retrospect. Third, the ground truth was manual volume quantification supervised by a senior radiologist which was the standard-of-reference in most previous reports, which however, is less accurate compared to volumetry of explanted organs. However, the goal of this study was to show that results of automated volumetry software and those of manual volume assessment by an experienced senior radiologist are almost perfectly comparable at this point, the comparison between automated CT liver volumetry and volumetry of explanted organs would be another interesting topic to investigate.

In conclusion, the results of fully automated liver volume quantification using this approved software are accurate and comparable with those of manual liver volume quantification and proved confident even if unenhanced lower-dose CT, thick-slice image data sets were used making them, thus, applicable for almost any examinational protocol. Future developments should aim at fully automated, accurate liver volume quantification, performed separately on an external server and subsequently transferring the results to the PACS.

## Supporting information

S1 File (SAV)

## Abbreviations

IQR: Interquartile range
MSL: Marginal space learning
PBT: Probabilistic boosting tree
SW: Spreading width
TPS: Thin plate spline
